# A New Antimicrobial Phenylpropanol from the Leaves of *Tabernaemontana inconspicua* Stapf. (Apocynaceae) Inhibits Pathogenic Gram-Negative Bacteria

**DOI:** 10.3390/antibiotics11010121

**Published:** 2022-01-17

**Authors:** Lidwine Ngah, Willifred Dongmo Tékapi Tsopgni, Judith Caroline Ngo Nyobe, Alain Tadjong Tcho, Moses K. Langat, Jean Claude Ndom, Eduard Mas-Claret, Nicholas John Sadgrove, Alain François Kamdem Waffo, Methee Phumthum

**Affiliations:** 1 Faculty of Sciences, Department of Chemistry, University of Douala, Douala P.O. Box 24157, Cameroon; lidwingah@yahoo.fr (L.N.); willifred2kpi@yahoo.fr (W.D.T.T.); ndomjefr@yahoo.com (J.C.N.); akamdemfr@yahoo.fr (A.F.K.W.); 2 Laboratory of Quality Control for Food, Pharmaceutical and Cosmetic Products, Department of Thermal Engineering and Energy, University Institute of Technology, University of Douala, Douala P.O. Box 8698, Cameroon; njudithcaroline@yahoo.fr; 3 Department of Chemistry, Faculty of Sciences, University of Buea, Buea P.O. Box 63, Cameroon; alainstone1@yahoo.fr; 4 Royal Botanic Gardens, Kew, Kew Green, Richmond, Surrey TW9 3AE, UK; m.langat@kew.org (M.K.L.); e.mas-claret@kew.org (E.M.-C.); n.sadgrove@kew.org (N.J.S.); 5 Department of Pharmaceutical Botany, Faculty of Pharmacy, Mahidol University, Bangkok 10400, Thailand

**Keywords:** *Tabernaemontana inconspicua*, Apocynaceae, alkaloids, antimicrobial, irisdichototin G

## Abstract

A chemical investigation of the leaves of *Tabernaemontana inconspicua* Stapf. led to the isolation of a new phenylpropanol derivative, namely irisdichototin G (**1**), together with nine known compounds, including one polyol derivative, dambonitol (**2**); three alkaloids, 10-hydroxycoronaridine (**3**), voacristine (**4**) and vobasine (**5**); two triterpenes lupeol (**6**), betulinic acid (**7**) and three sterols, sitosterol (**8**), sitosterol-3-O-β-D-glucopyranoside (**9**) and stigmasterol (**10**). The structure of the new compound, as well as those of the known ones, was established by means of spectroscopic methods: NMR analysis (^1H^ and ^13^C NMR, 1H-1H-COSY, HSQC, HMBC and NOESY), high-resolution mass spectrometry (HR-ESI-MS) and comparisons with previously reported data. Among the known compounds, compound **2** was firstly reported from the family Apocynaceae. Compounds **1**–**5** were tested for their antimicrobial effects against three Gram-negative organisms associated with human wound and systemic infections, namely *Haemophilus influenzae* 9435337A, *Klebsiella pneumoniae* 17102005 and *Pseudomonas aeruginosa* 2137659B. Compounds **1**, **3**, and **5** showed significant antimicrobial effects with minimum inhibitory concentrations (MIC) of 62.5 μg/mL, 62.5 μg/mL and 7.81 μg/mL, respectively, against *Haemophilus influenzae*, whereas compounds **1** and **5** showed significant antimicrobial effects, with a MIC value of 31.25 μg/mL against *Pseudomonas aeruginosa*. In addition, compound **3** showed significant antimicrobial activity, with a MIC value of 31.25 μg/mL against *Klebsiella pneumoniae*.

## 1. Introduction

*Tabernaemontana* is one out of 415 genera in the family Apocynaceae, distributed throughout the tropical world, in some subtropical regions, and of course parts of Africa and Asia. It consists of about 110 species, including the species of the current study, namely *Tabernaemontana inconspicua* Stapf. [1], which is a shrub with green or yellow to orange bark. The different organs from species in the genus *Tabernaemontana* are used in African traditional medicine as local anesthetics, for aphrodisiac applications and as purgatives [2,3]. In scientific studies, the extracts from species in *Tabernaemontana* confer significant biological effects across a wide range of bioassays, such as antioxidation, cytotoxicity, antimicrobial and antiparasitic activities [2,4]. Many classes of secondary metabolites have been reported from this genus, with alkaloids as their main class of compound. Some of these alkaloids are coronaridine, 5,6-dioxo-11-hydroxy voacangine [5], ibogamine16-carboxylic acid-17,20-didehydro-5,6-dioxo-10-methoxy-methyl ester [6], voacangine [7], and perakine [8]. The roots and stem bark of *T. inconspicua* contain alkaloids, such as 5,6-dioxo-11-methoxy voacangine and (-)-apparicine-21-one [9]. In addition, triterpenoids, steroids, and ceramides were also reported [10]. In continued research of bioactive compounds from Central African flora, the investigation focused on the leaves and isolated a new phenylpropanol derivative, namely irisdichototin G (**1**) together with nine known compounds, including dambonitol (**2**) reported for the first time from the family Apocynaceae.

*Tabernaemontana inconspicua* Stapf. is a shrub with green or yellow to orange bark. This species is an Africa endemic that is currently distributed in almost all tropical countries [1]. It grows up to 15 m tall and 6 m wide. The plant has not been adequately studied for its phytochemistry and biological activities. Only a few studies revealed that the plant contains indole alkaloids, which have cytotoxic activities [10]. The aim of the study was to elucidate the phytochemistry and antimicrobial activity of leaf extracts from *T. inconspicua*.

## 2. Materials and Methods

### 2.1. General Experimental Procedures

Thin-layer chromatography was performed using Merck TLC Silica gel 60 F254 or TLC Silica gel 60 RP-18 F254S. UV light (254 nm and 354 nm) and/or a 10 % H_2_SO_4_ stain were used to visualize the spots on TLCs. Column chromatography was performed on silica gel provided by Brunschwig (32–63 mesh, 60Å) prepacked columns. NMR measurements were carried out on a Bruker Avance III HD 500 MHz spectrometer (^1H^: 500 MHz, ^13^C: 125 MHz). Deuterated solvents were obtained from Cambridge Isotope Laboratories. HRESI-MS was performed on a MicrOTOF-Q mass spectrometer (Bruker, Germany). ESI-MS reaction monitoring was carried out using a Bruker esquire HCT Ion trap mass spectrometer. IR spectra were recorded on a Bruker FT-IR Tensor II using a Golden Gate diamond ATR system. Optical rotations were measured on a Perkin Elmer Polarimeter 241 using the sodium lamp (589 nm) and a 10 cm long cuvette. Microwave heating was performed on a Biotage Initiator Microwave using Biotage microwave vials. UV/VIS spectra were recorded on a UV/VIS Lambda 25,190–1100 nm. Irradiations were performed using Rayonet photochemical reactors. 

### 2.2. Plant Material

The leaves of *T. inconspicua* were collected in daylight during October 2019 at Nlong locality (3°31′10.8′′ N, 11°6′11.89′′ E), in the Central region of Cameroon. The plant was identified by Mr. Victor Nana, botanist at the National Herbarium of Cameroon, where a specimen was deposited under the voucher number NHC 61026. 

### 2.3. Extraction and Isolation

The air-dried and powdered leaves (1.4 kg) of *T. inconspicua* were soaked twice, using methanol for 48 h and 24 h, respectively. The solvent was evaporated using a rotary-evaporator to afford crude extracts and a yield of 65.8 g was determined, of which a portion was used in silica gel column chromatography. The mobile phase used ethyl acetate (EtOAc) in hexane (Hex), following a gradient from 05:95 to 100:00 (*v*/*v*), respectively. Then, 100 mL volumes were collected in chromatography and pooled based on their TLC profiles into 7 sub-fractions (F1–F7). The mixture of β-sitosterol (**8**) and stigmasterol (41.05 mg) (**10**) precipitated as a white powder after recrystallization of F2 (145.10 mg, Hex–EtOAc (9:1, *v*/*v*)), as well as β-sitosterol-3-O-β-D-glucopyranoside (**9**) (92.40 mg) from F7 (250.35 mg, Hex–EtOAc (3:7, *v*/*v*)). F1 (210.50 mg, Hex–EtOAc (19:1, *v*/*v*)) followed the same treatment to give lupeol (**6**), whilst F3 (110.50 mg, Hex–EtOAc (17:3, *v*/*v*)) was further chromatographed on silica gel with an isocratic solvent system of Hex–EtOAc (9:1, *v*/*v*) to give betulinic acid (**7**) (11.25 mg). F4 (85.55 mg, Hex–EtOAc (8:2, *v*/*v*)) was further chromatographed on sephadex LH-20 eluted with methanol to afford vobasine (**5**) (17.30 mg). By the same means, 10-hydroxycoronaridine (**3**) (7.10 mg) and voacristine (**4**) (6.10 mg) were obtained from F5 (145.20 mg, Hex–EtOAc (7:3, *v*/*v*)). In addition, F6 (35.40 mg, Hex–EtOAc (1:1, *v*/*v*)) was purified on silica gel column chromatography with an isocratic elution using the solvent system of Hex–EtOAc (3:2, *v*/*v*) to afford compound **1** (10.60 mg) and dambonitol (**2**) (15.80 mg).

### 2.4. Spectroscopy Data of Compound ***1***

(1β, 2β)-1-(3-Hydroxy-4-methoxyphenyl)propane-1,2,3-triol with the given name irisdichototin G: brown oil; HRESIMS at *m*/*z* 237.0731 [M+Na]+ (calc. For C_10_H_14_O_5_Na *m*/*z* 237.0720). ^1H^ and ^13^C NMR data; see Table 1.

### 2.5. Antimicrobial Effects

Compounds **1**–**5** and the crude extract were tested for their antimicrobial effects against *Haemophilus influenzae* 9435337A, *Klebsiella pneumoniae* 17102005 and *Pseudomonas aeruginosa* 2137659B. The organisms were chosen based on their roles in human infection, and because they are Gram-negative. The latter is to represent pathogens that are otherwise poorly represented in antimicrobial research of natural products. The method followed the alamar blue method described by Collins and Franzblau [11], with Levofloxacin as a positive control and no treatment as the negative control. Briefly, a two-fold serial broth dilution was conducted in a 96-well microtiter plate, with a starting concentration of 250 µg·mL^−1^ and diluting across 10 wells. The plate was inoculated (giving final concentrations of treatments at 250–0.5 µg·mL^−1^) and organisms were grown overnight and then stained using the alamar blue reagent, with the appearance of indigo as an indicator of growth, and no color as no growth. The MIC and MBC values are presented as an average of three replicates.

## 3. Results and Discussion

**Figure 1:**
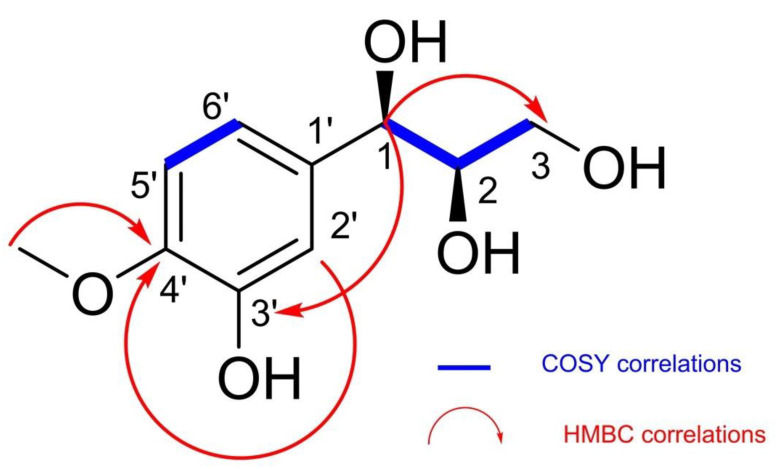
Key 1H-1H COSY and HMBC correlations of compound **1**.

**Figure 2:**
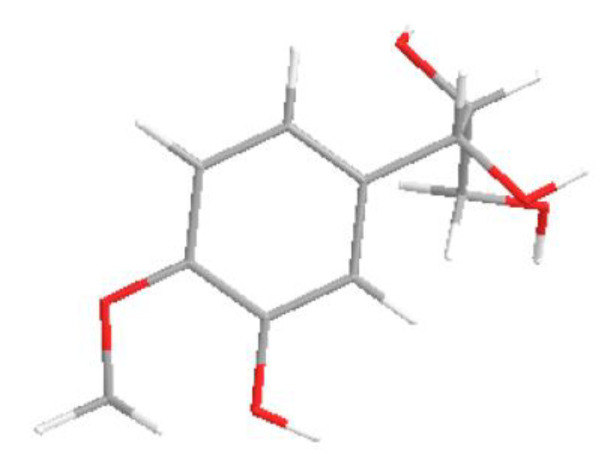
Key NOESY correlation of compound **1**.

Compound **1** (Figure 1) was obtained as a brown oil and gave a positive ferric chloride test, indicating its phenolic nature. Its molecular formula C_10_H_14_O_5_, implying four degrees of unsaturations, was determined from its HR-ESIMS spectrum, which showed, in positive mode, the sodium adduct ion peak [M + Na]^+^ at *m*/*z* 237.0731 (calc. For C_10_H_14_O_5_Na *m*/*z* 237.0720). The ^1H^ NMR spectrum of **1** showed signals for an ABX system at δH 7.02 (1H, d, J = 2.0), 6.80 (1H, dd, J = 8.0; 2.0) and 6.71 (1H, d, J = 8.0), indicating a 1,2,4-trisubstituted benzene ring. In addition, it showed proton signals for oxymethines at δH 4.54 (1H, d, J = 6.2) and δ3.69 (1H, m) and those of diasteriotopic protons of oxymethylene at δH 3.69 (1H, m) and δ3.50 (1H, m), suggesting the presence of the propane-1,2,3-triol moiety in the structure of compound **1**. Finally, it displayed a signal for a methoxyl group at δH δ3.88 (3H, s). The ^13^C NMR of compound **1** supported the presence of a benzene ring with the corresponding carbon signals at δC 119.2 (C-6*′*), 114.3 (C-5*′*), 110.2(C-2*′*), 147.4 (C-4*′*), 133.5 (C-3*′*) and 104.0 (C-1*′*); it also supported the presence of propane-1,2,3-triol with the carbon signals at δC 76.2 (C-2) and 74.1 (C-1) for the oxymethynes and δC 62.9 (C-3) for the oxymethylene. Furthermore, it showed a carbon signal for a methoxyl group at δC 55.0. The HMBC spectrum showed cross correlation between the protons H-2*′* (δH 7.02), H-6*′* (δH 6.80), OCH3 (δH 3. 88) and the same carbon C-4*′* (δC 147.4), which allowed for the placement of the methoxyl group at C-4*′*. In addition, the correlation between the proton H-1 at δH 4.54 and carbons C-2 (δC 76.2), C-3 (δC 62.9), C-3*′* (δC 133.5) and C-1*′* (δC 104.0) allowed the placement of the propane-1,2,3-triol moiety at C-1*′*. The third substituent on the benzene ring was deduced as a hydroxyl group according to the molecular mass. The relative configuration of C-1 was deduced as β based on the coupling constants and the chemical shift value of the benzylic proton H-1 (δH 4.54, d, J = 6.2) [12] and that of C-2, confirmed by a correlation between H-1 and H-2 in the NOESY spectrum for a cis configuration (Figure 2). On the basis of all this evidence, the structure of compound **1** was deduced ass (1β, 2β)-1-(3-hydroxy-4-methoxyphenyl)propane-1,2,3-triol with the given name irisdichototin G.

The known compounds were identified as dambonitol (**2**) [13], three alkaloids, 10-hydroxycoronaridine (**3**) [14], voacristine (**4**) [14] and vobasine (**5**) [14], two triterpenes lupeol (**6**) [15] and betulinic acid (**7**) [16] and three sterols, sitosterol (**8**) [15], sitosterol-3-O-β-D-glucopyranoside (**9**) [15] and stigmasterol (**10**) [15] (Figure 3, Figures S1–S25)). Among these compounds, dambonitol (**2**) is reported for the first time in the family Apocynaceae. However, the three alkaloids reported herein are consistent with the known chemistry of Apocynaceae.

Compounds **1**–**5** and the crude extract were tested for their antimicrobial effects against *Haemophilus influenzae* 9435337A, *Klebsiella pneumoniae* 17102005 and *Pseudomonas aeruginosa* 2137659B. The result (Table 2) showed that, from the crude extract, compounds **1**, **3** and **5** exhibited significant antimicrobial effects with minimum inhibitory concentrations (MIC) of 15.625 μg/mL, 62.5 μg/mL, 62.5 μg/mL and 7.81 μg/mL, respectively, against *H. influenzae* and a bactericidal effect each, with an MBC/MIC ratio ≤ 4. In addition, the crude extract, compounds **1** and **5** showed significant antimicrobial effects with MIC values of 62.5 μg/mL, 31.25 μg/mL, and 31.25 μg/mL, respectively, against *P. aeruginosa* and a bactericidal effect each, with an MBC/MIC ratio ≤ 4. Furthermore, the crude extract and compound **3** showed significant antimicrobial effects with a MIC of 31.25 μg/mL against *K. pneumoniae* and a bactericidal effect each, with an MBC/MIC ratio ≤ 4. Compounds **2** and **4** were found to be inactive against the three strains. These results show that compound **3** may be the one responsible for the activity of the crude extract and the synergistic effect of compound **3** by other compounds in the crude extract is not evident.

In the research of natural products, it is more common to find compounds that are active against Gram-positive organisms, such as *Staphylococcus aureus*, among others [17]. This is because the cell walls of Gram-negative organisms are fortified by a hydrophilic periplasmic space that makes it difficult for lipophilic compounds to enter the cell. However, in the current study, the compounds that were active had a moderately high polar head space, caused by the presence of hydroxyl groups, which increase aqueous solubility and the ability to traverse the cell walls of Gram-negative bacteria. Out of the active compounds, two major chemical classes are represented, i.e., the phenylpropanoids, and indole alkaloids (vinca and vobasan parent groups). This indicates the likelihood that different mechanisms of activity are possible. The vinca alkaloids are associated with a wide range of biological effects, but in the context of mammalian cells, they inhibit microtubule formation and prevent successful mitosis [18], but this is unlikely to be related to their mechanism in bacteria, since bacteria do not have nuclei. Hence, the mechanisms need to be investigated independently. Regarding the phenylpropanoids, it is well known that small aromatic compounds disrupt the cell wall barrier in both Gram-positive and Gram-negative bacteria [19], so this should be investigated as a possible mechanism for compound **1** of the current study.

## 4. Conclusions

This research led to the isolation of a new phenylpropanol derivative namely irisdichototin G (**1**) together with dambonitol (**2**). The latter is reported herein for the first time in the family Apocynaceae. Three known alkaloids were also reported that are commonly reported in Apocynaceae. Furthermore, compounds, **1**, **3** and **5** showed significant antimicrobial effects against the Gram-negative organisms, *Haemophilus influenzae* 9435337A, *Klebsiella pneumoniae* 17102005 and *Pseudomonas aeruginosa* 2137659B, with MIC values ranging from 7.8 to 125 μg/mL and bactericidal effects ranging from two-fold to four-fold differences to MIC values. The limitations of the current study are that the antimicrobial effects can only be achieved if the extracts are applied topically, because the oral consumption of the plant cannot produce systemic concentrations high enough to meet the necessary MIC concentrations. However, the study demonstrates that the extracts of this plant are significant in the context of topical disinfection of Gram-negative bacteria.

## Figures and Tables

**Figure 3:**
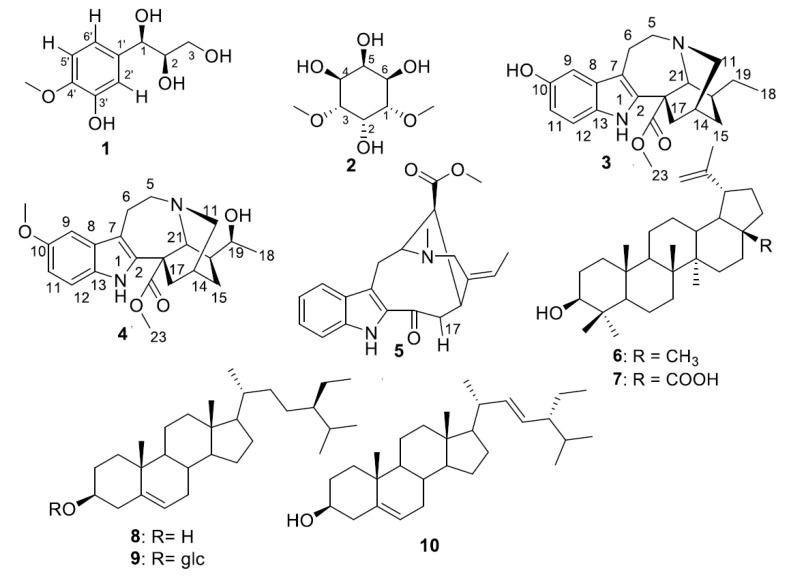
Chemical structures of compounds **1**–**10** from *T. inconspicua*.

**Table 1:** ^1H^ (500 MHz) and ^13^C NMR (125 MHz) data for compound (**1**) in MeOD.

Position	δC	δH (Mult.; J)
1	74.1	4.54 (1H, d, J = 6.2)
2	76.2	3.69 (1H, m)
3	62.9	3.69 (1H, m)3.50 (1H, m)
1′	104.0	/
2′	110.2	7.02 (1H, d, J = 2.0)
3′	133.5	/
4′	147.4	/
5′	114.3	6.71 (1H, dd, J = 8.0 ; 2.0)
6′	119.2	6.80 (1H, d, J = 8.0)
CH3O-	55.0	3.88 (1H, m)

**Table 2:** Average inhibitory and bactericidal concentrations (MIC and MBC) of the crude extract and compounds **1**–**5**.

Samples	Inhibitory Parameters (µg/mL)								
	*Haemophilus influenzae* 9435337A			*Klebsiella pneumoniae* 17102005			*Pseudomonas aeruginosa* 2137659B		
	MIC	MBC	MBC/MIC	MIC	MBC	MBC/MIC	MIC	MBC	MBC/MIC
Crude Extract	15.625	62.5	4	31.25	125	4	62.5	125	2
1	62.5	125	2	125	250	2	31.25	>250	ND
2	>250	>250	ND	>250	>250	ND	>250	>250	ND
3	62.5	125	2	125	250	2	250	>250	ND
4	>250	>250	ND	>250	>250	ND	>250	>250	ND
5	7.81	31.25	4	31.25	125	4	31.25	125	4
Levofloxacin	1.95	7.81	4	0.48	1.95	4	0.48	1.95	4

ND: not determined; MIC = Minimum inhibitory concentration; MBC = Minimum bactericidal concentration; The ratio MBC/MIC determine the bactericidal (MBC/MIC ≤ 4) or bacteriostatic (MBC/MIC > 4) effects of extracts. The activity of plant extract and compounds will be classified as significant (MIC < 100 µg/mL), moderate (100–625 µg/mL), or weak (MIC > 250 µg/mL).

## Data Availability

Not applicable.

## Supplementary Materials

The following are available online at https://www.mdpi.com/article/10.3390/antibiotics11010121/s1, Figures S1–S25: Mass spectra and NMR of all isolated compounds.
