# Supplementary material for: A New Antimicrobial Phenylpropanol from the Leaves of Tabernaemontana inconspicua Stapf. (Apocynaceae) Inhibits Pathogenic Gram-Negative Bacteria
Source: Antibiotics (Basel). 2022 Jan 17;11(1):121. doi: 10.3390/antibiotics11010121 (PMC8773313; doi:10.3390/antibiotics11010121)
Supplement: Supplementary file 1 [file antibiotics-11-00121-s001.zip › antibiotics-1547593-supplementary.pdf]

A New Antimicrobial Phenylpropanol from the Leaves of *Tabernaemontana inconspicua* Stapf. (Apocynaceae)

Lidwine Ngah [1], Willifred Dongmo Tékapi Tsopgni [1], Judith Caroline Ngo Nyobe [2], Alain Tadjong Tcho [3], Moses K Langat [4], Jean Claude Ndom [1], Eduard Mas-Claret [4], Nicholas John Sadgrove [4], Alain François Kamdem Waffo [1] and Methee Phumthum [4, 5, *]

[1] Department of Chemistry, University of Douala, Faculty of Sciences, 24157 Douala, Cameroon
[2] Laboratory of Quality Control for Food, Pharmaceutical and Cosmetic Products, Department of Thermal Engineering and Energy, University Institute of Technology, University of Douala, PO Box: 8698 Douala, Cameroon
[3] Department of Chemistry, University of Buea, Faculty of Sciences, PO Box: 63 Buea, Cameroon
[4] Royal Botanic Gardens, Kew, Kew Green, Richmond, Surrey, TW9 3AE, UK
[5] Department of Pharmaceutical Botany, Faculty of Pharmacy, Mahidol University, Bangkok, 10400, Thailand

Contents:

Figure S1: (+)-HR-ESI spectrum of **1** ………………………..……………………………….. …2
Figure S2: ^{1}H NMR spectrum (500 MHz) of **1** in MeOD …………………………………….... .2
Figure S3: ^{13}C NMR spectrum (125 MHz) of **1** in MeOD ……………...……………………... .3
Figure S4: DEPT spectrum (125 MHz) of **1** in MeOD ……………………………………………... .3
Figure S5: HMBC spectrum of **1** in MeOD …………………………………………………….... .3
Figure S6: NOESY spectrum of **1** in MeOD …………………………………………………….... .4
Figure S7: ^{1}H NMR spectrum (500 MHz) of **2** in MeOD …………………………………….... .4
Figure S8: ^{1}H-^{1}H COSY spectrum of **2** in MeOD ……………..……………….……………….... .5
Figure S9: ^{13}C NMR spectrum (125 MHz) of **1** in MeOD ……………...……………………... .5
Figure S10: DEPT spectrum (125 MHz) of **1** in MeOD …….……………………………………... .6
Figure S11: HMBC spectrum of **2** in MeOD ……………………….…………………………….... .6
Figure S12: ^{1}H NMR spectrum (500 MHz) of **3** in MeOD …………………………………….... .7
Figure S13: ^{1}H-^{1}H COSY spectrum of **3** in MeOD ….………..…………….…………………... ..7
Figure S14: ^{13}C NMR spectrum (125 MHz) of **3** in MeOD ………………...…………………... ..8
Figure S15: DEPT spectrum (125 MHz) of **3** in MeOD ………………………………………….... ..8
Figure S16: HMBC spectrum of **3** in MeOD ……………………………………………………..... ..9
Figure S17: ^{1}H NMR spectrum (500 MHz) of **4** in MeOD …………………………………….... .9
Figure S18: DEPT spectrum (125 MHz) of **4** in MeOD ………………………………………... .10
Figure S19: HMBC spectrum of **4** in MeOD …………………………………………………..... ..10
Figure S20: ^{1}H NMR spectrum (500 MHz) of **5** in MeOD …….………………………………... 11
Figure S21: HMBC spectrum of **5** in MeOD ……………………….………………………….... 11
Figure S22: ^{1}H NMR spectrum (500 MHz) of **6** in $CDCl_3$ ……………………………………... .12
Figure S23: ^{1}H NMR spectrum (500 MHz) of **7** in MeOD ……..…………………….... ………..12
Figure S24: ^{1}H NMR spectrum (500 MHz) of **8** and **10** in $CDCl_3$…………………….. …………...13
Figure S25: ^{1}H NMR spectrum (500 MHz) of **9** in $CDCl_3$ / MeOD …….…………………….. ...13

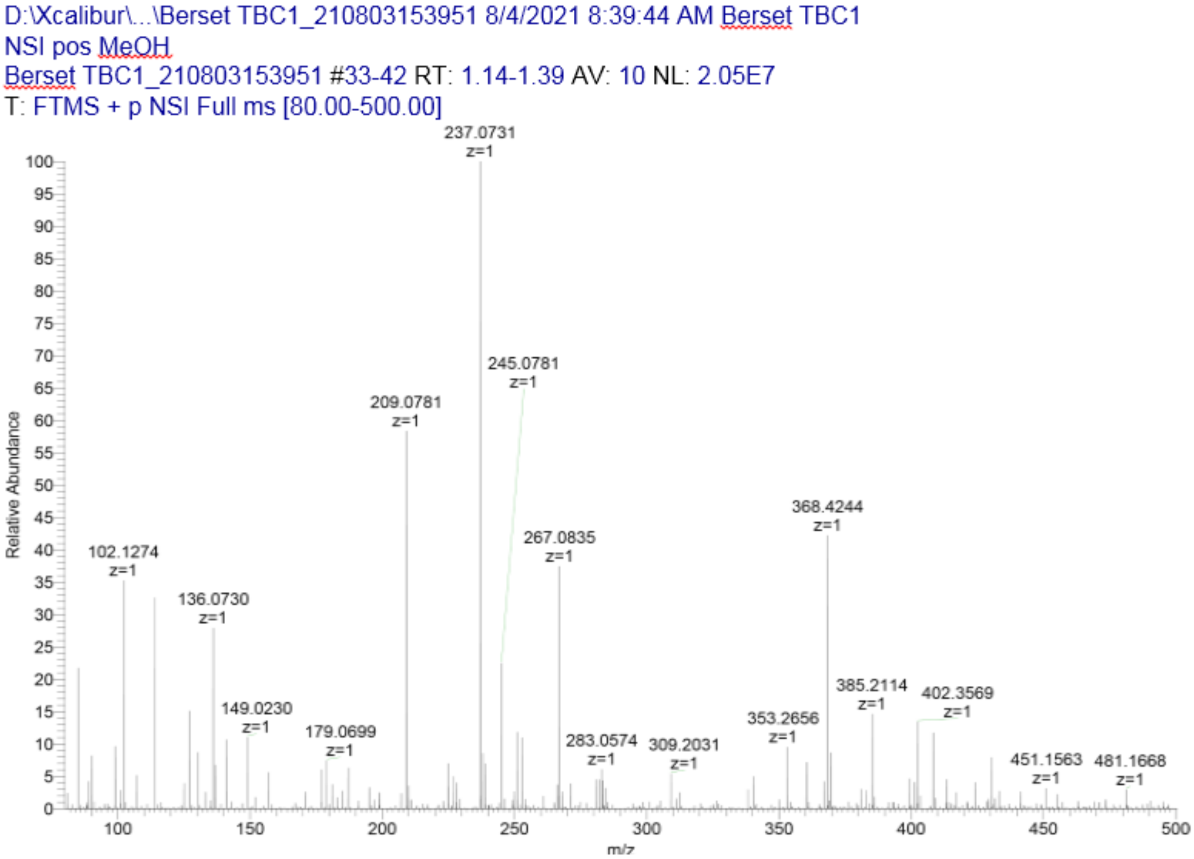

Figure S1: ^{1}H NMR spectrum (500 MHz) of **1** in MeOD

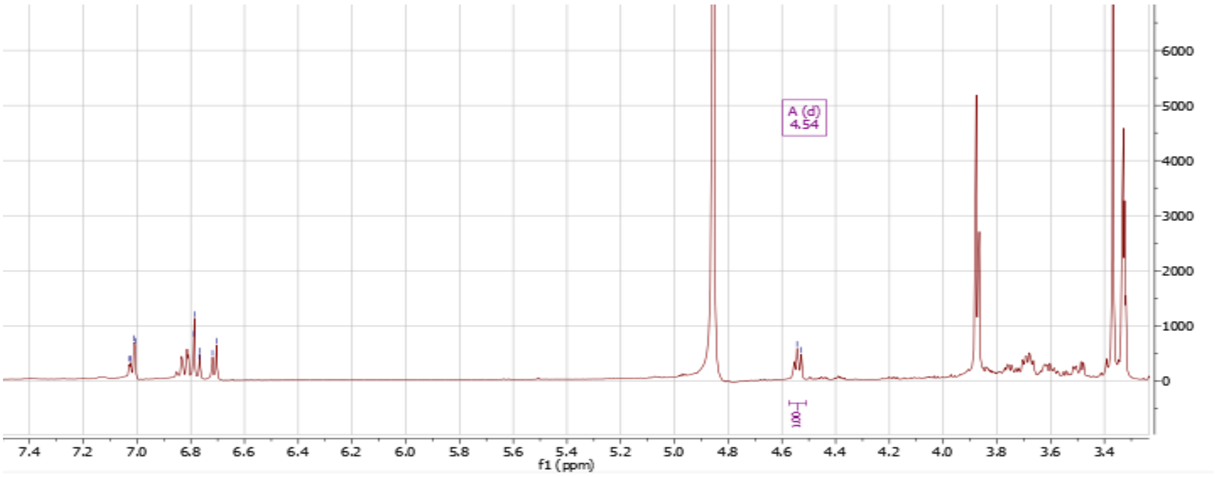

Figure S2: ^{1}H NMR spectrum (500 MHz) of **1** in MeOD

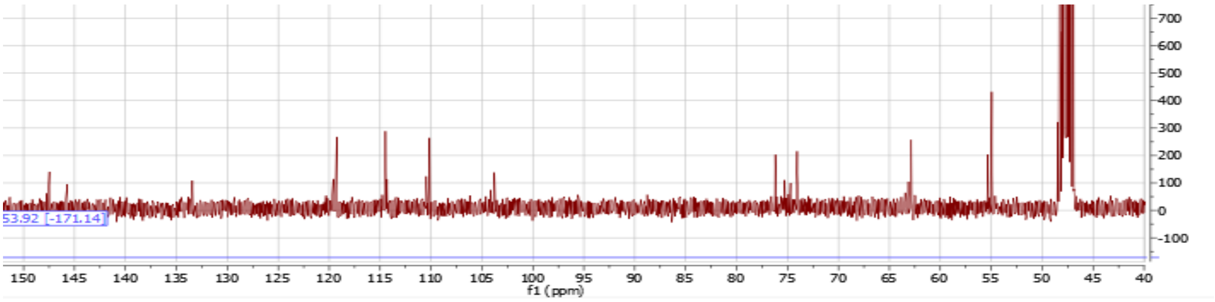

Figure S3: ^{13}C NMR spectrum (125 MHz) of **1** in MeOD

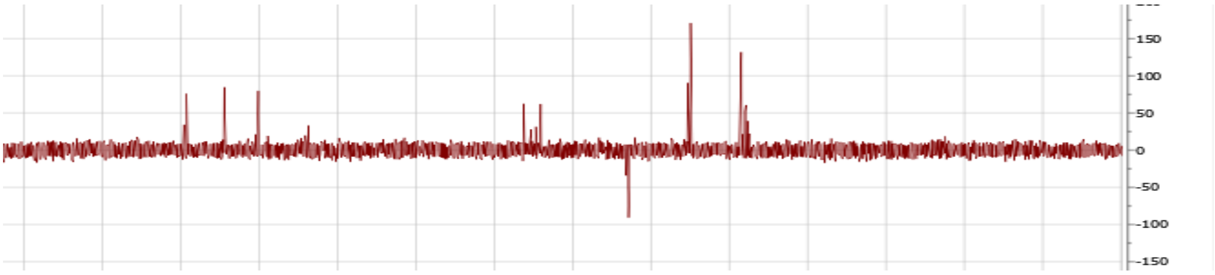

Figure S4: DEPT 135 spectrum (125 MHz) of **1** in MeOD

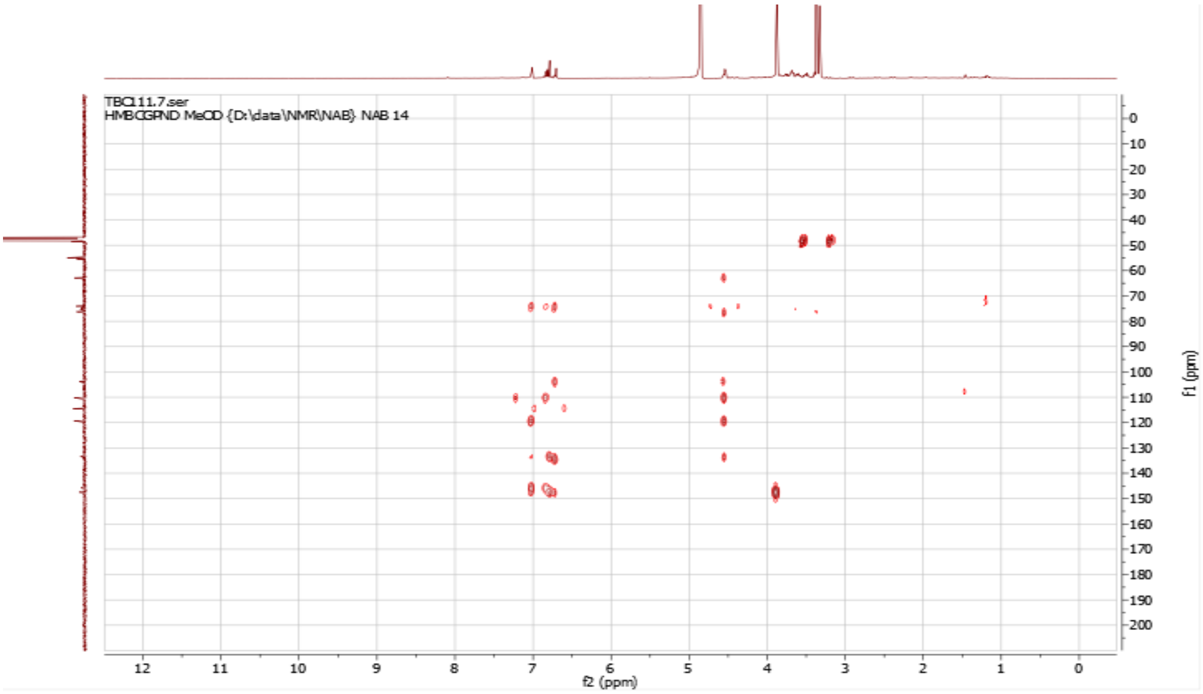

Figure S5: HMBC spectrum of **1** in MeOD

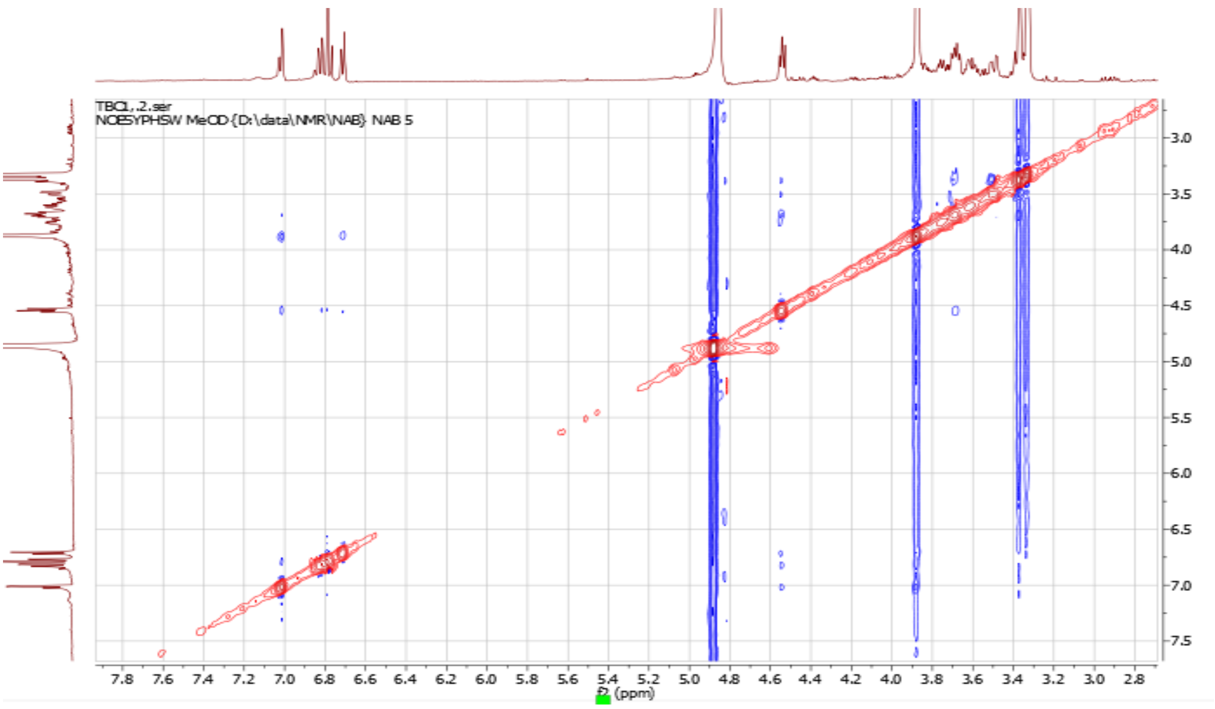

Figure S6: HMBC spectrum of **1** in MeOD

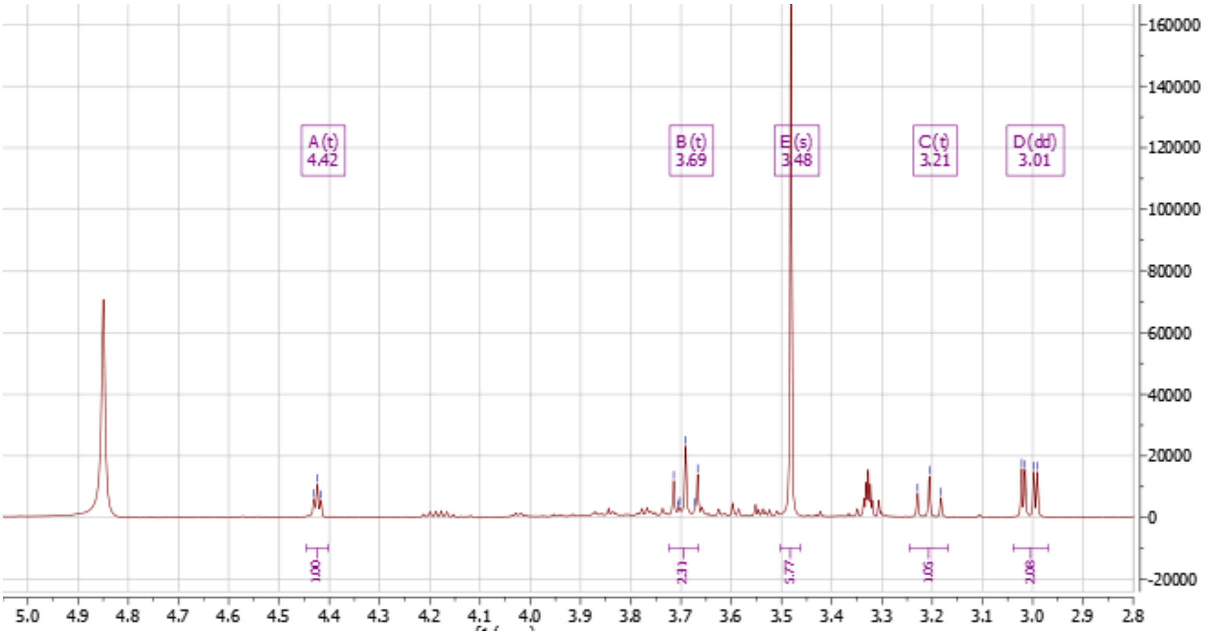

Figure S7: ^{1}H NMR spectrum (500 MHz) of **2** in MeOD

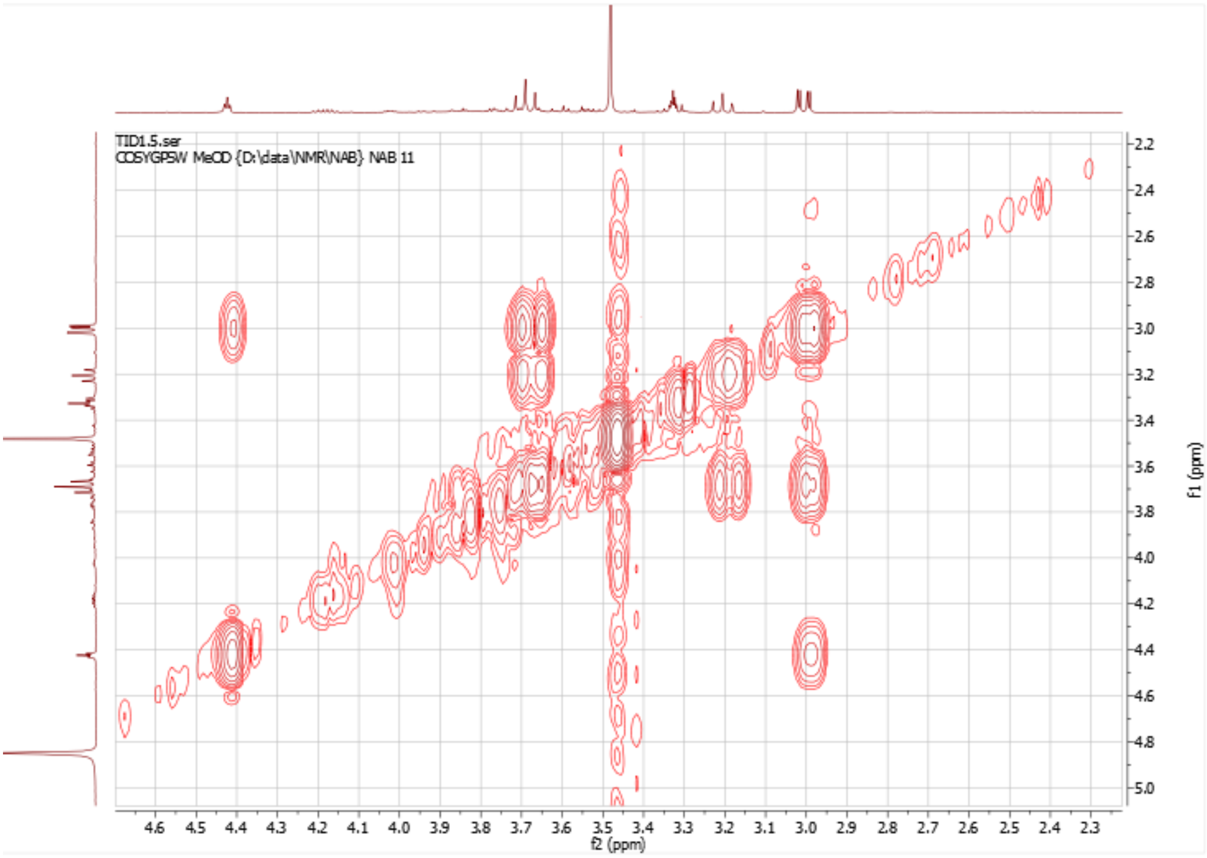

Figure S8: COSY ^{1}H-^{1}H spectrum (500 MHz) of **2** in MeOD

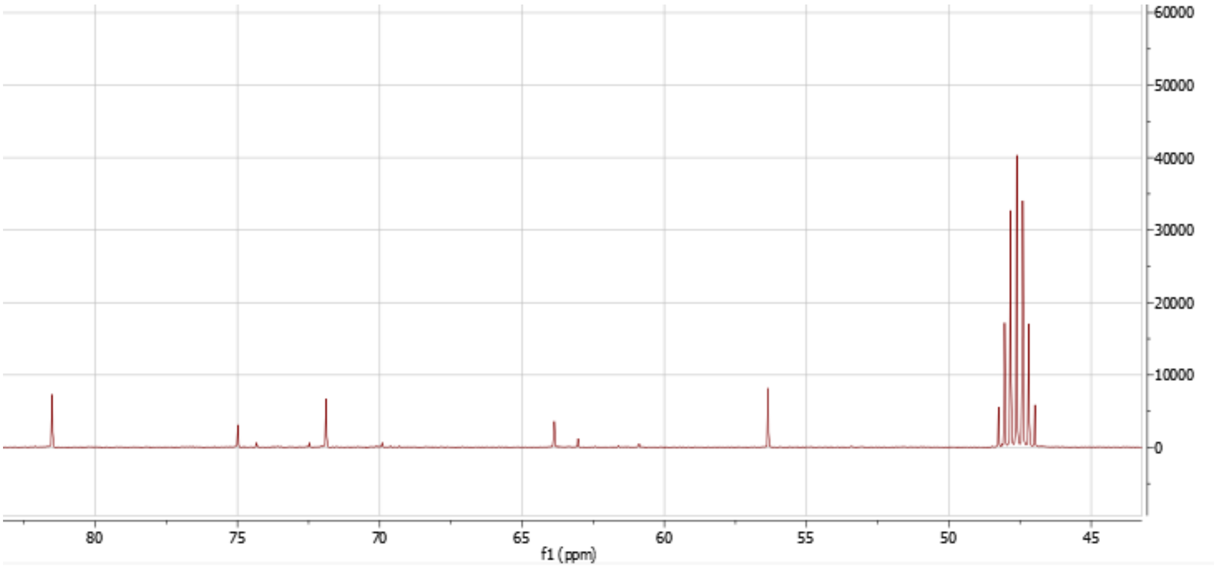

Figure S9: ^{13}C NMR spectrum (125 MHz) of **2** in MeOD

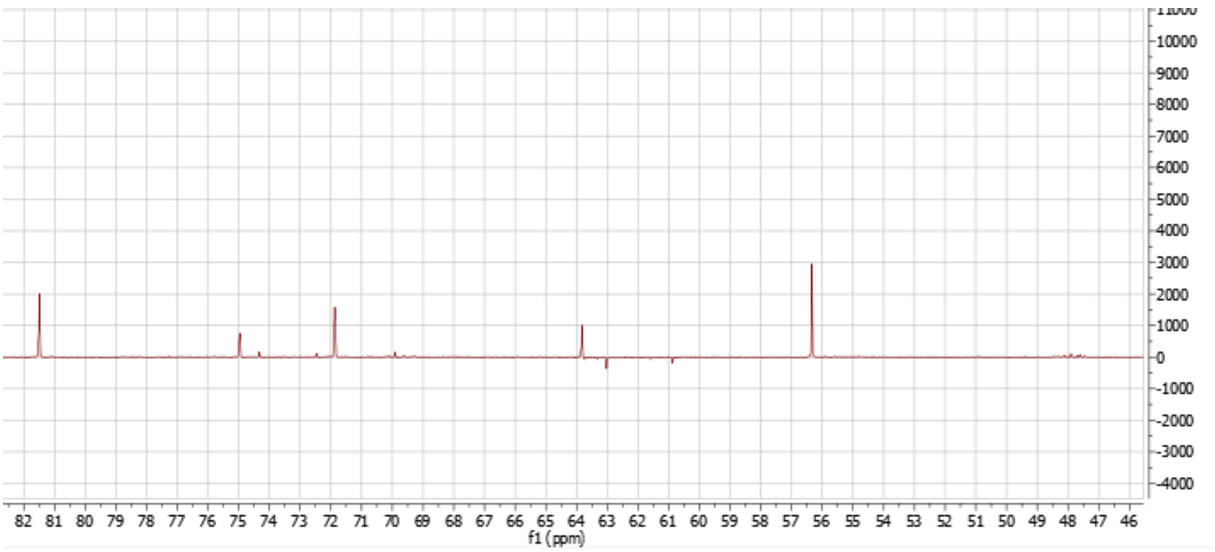

Figure S10: DEPT 135 spectrum (125 MHz) of **2** in MeOD

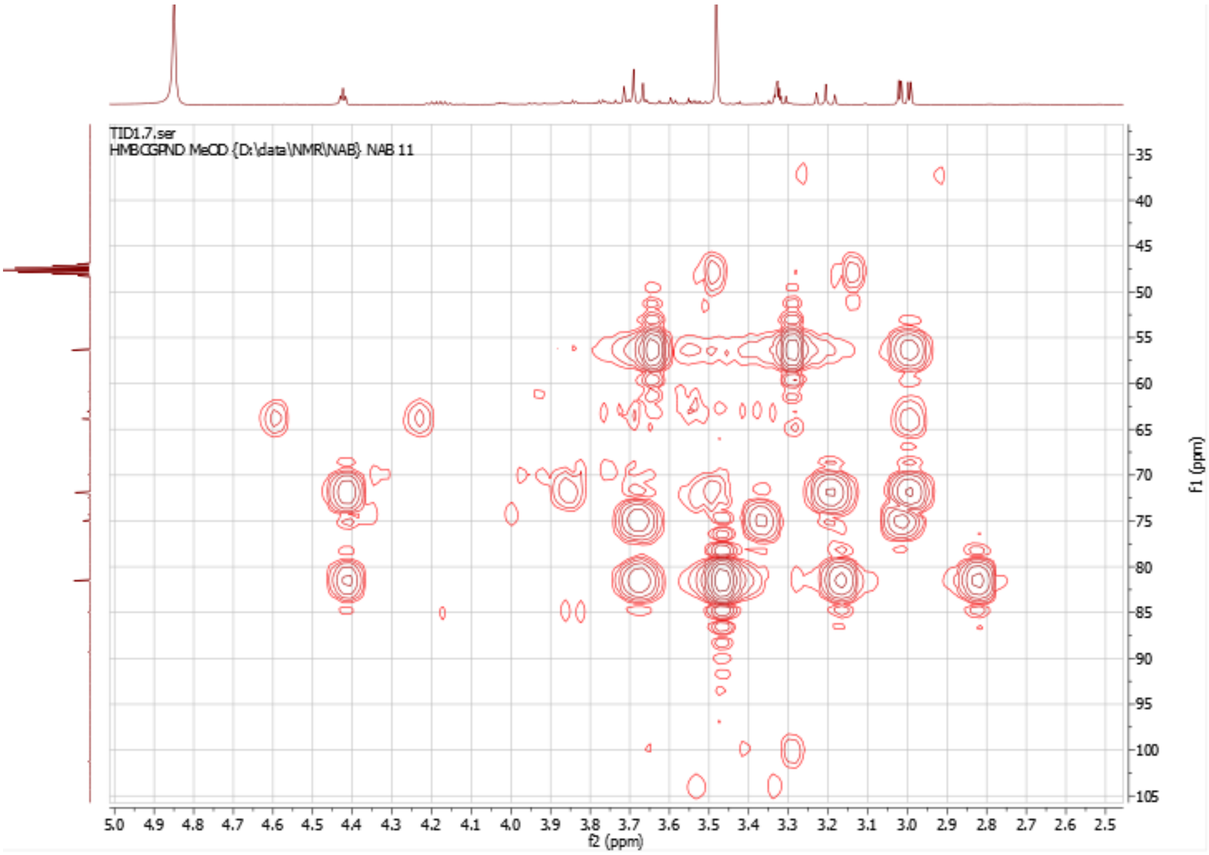

Figure S11: HMBC spectrum of **2** in MeOD

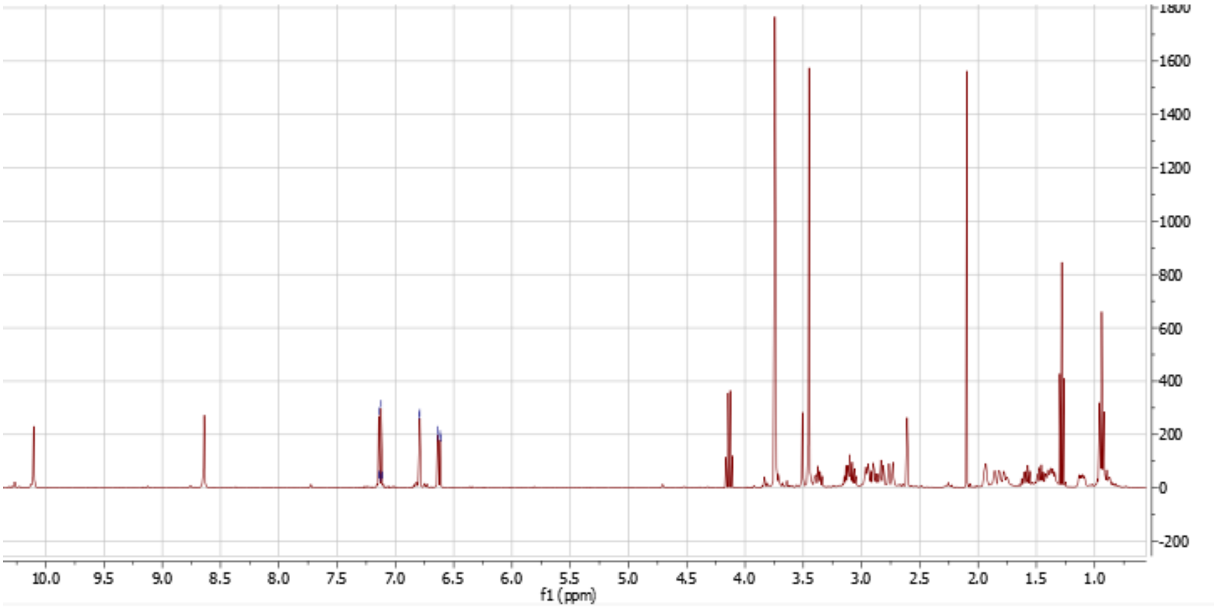

Figure S12: ^{1}H NMR spectrum (500 MHz) of **3** in MeOD

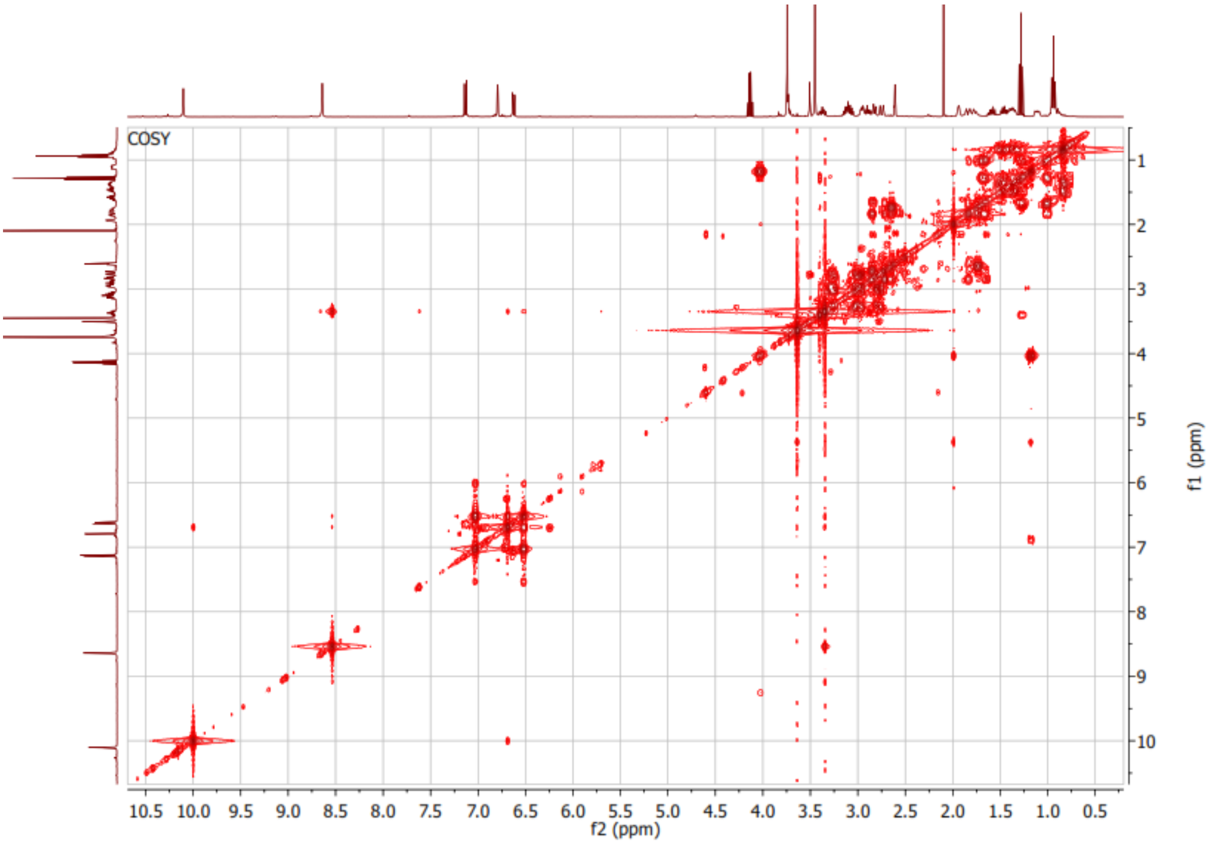

Figure S13: COSY ^{1}H-^{1}H spectrum (500 MHz) of **3** in MeOD

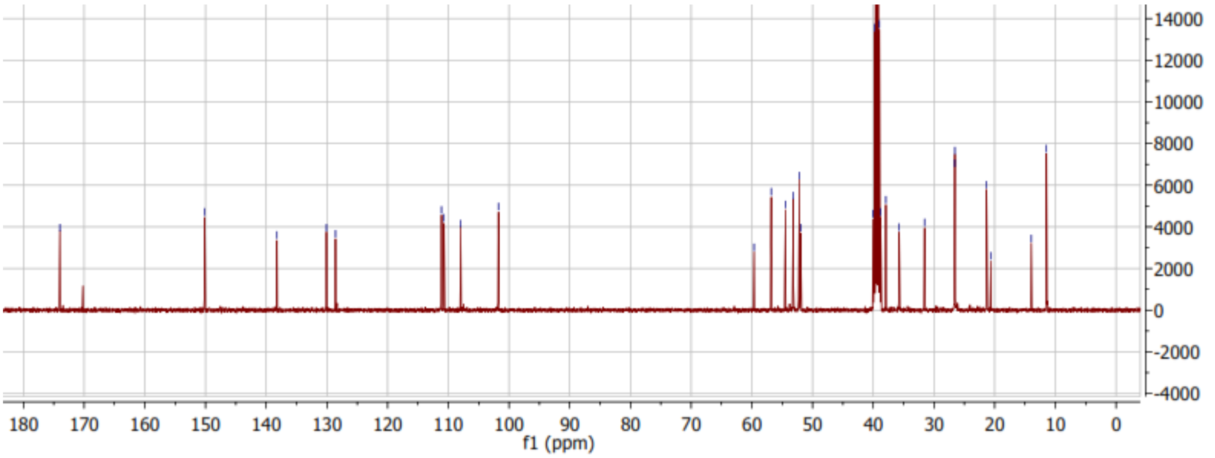

Figure S14: ^{13}C NMR spectrum (125 MHz) of **3** in MeOD

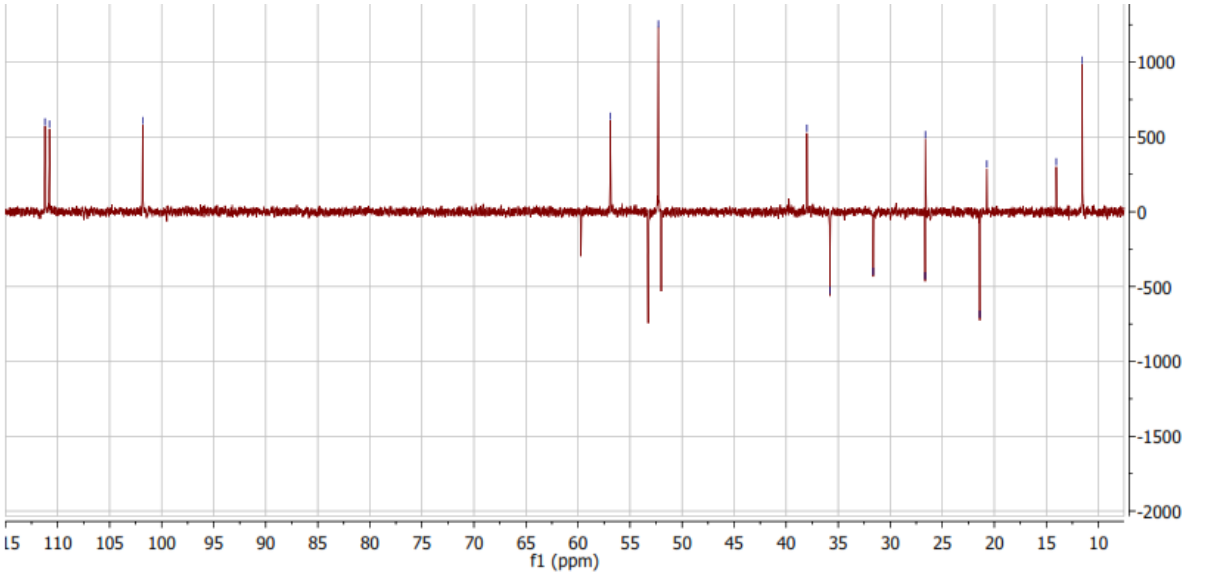

Figure S15: ^{13}C NMR spectrum (125 MHz) of **3** in MeOD

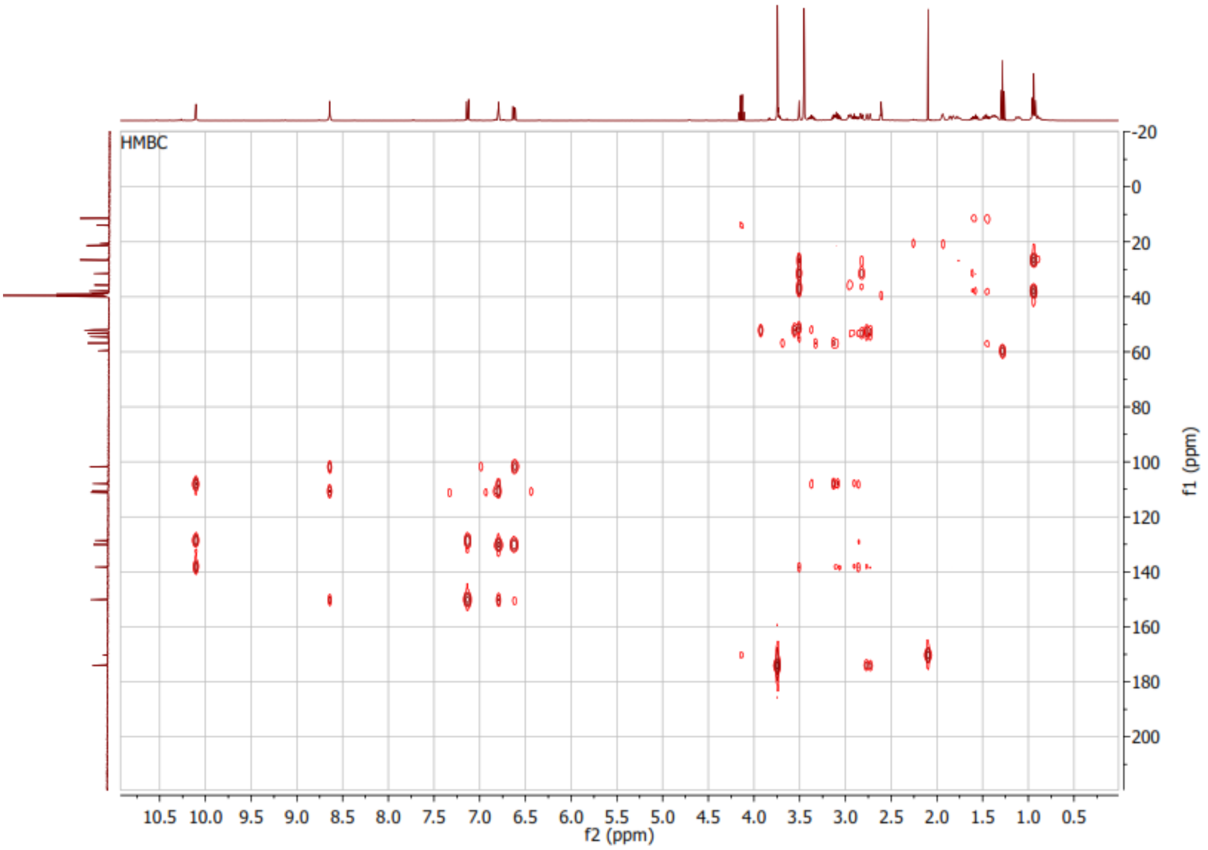

Figure S16: HMBC spectrum of **3** in MeOD

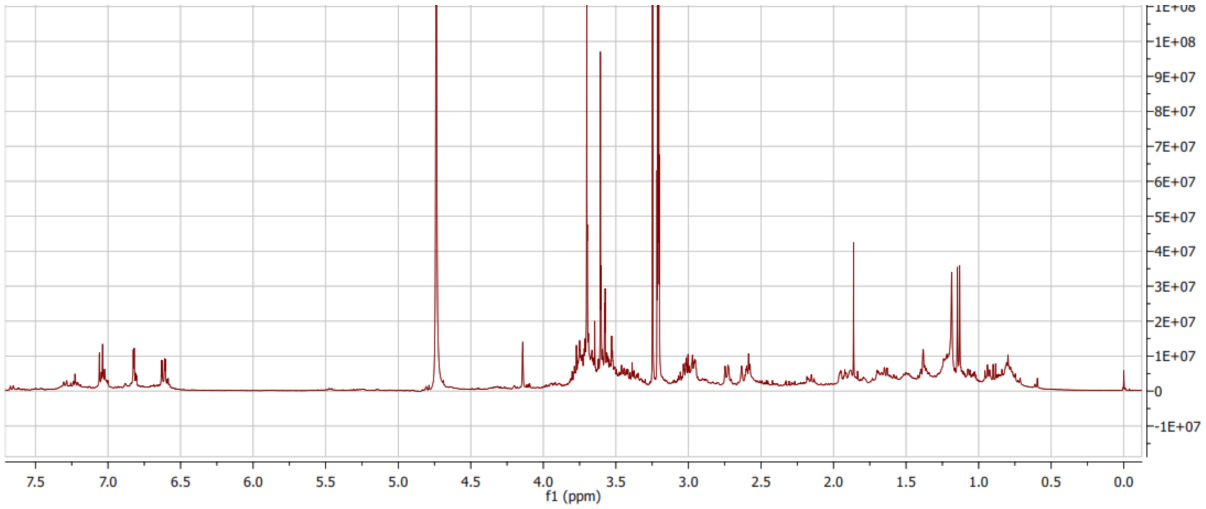

Figure S17: ^{1}H NMR spectrum (500 MHz) of **4** in MeOD

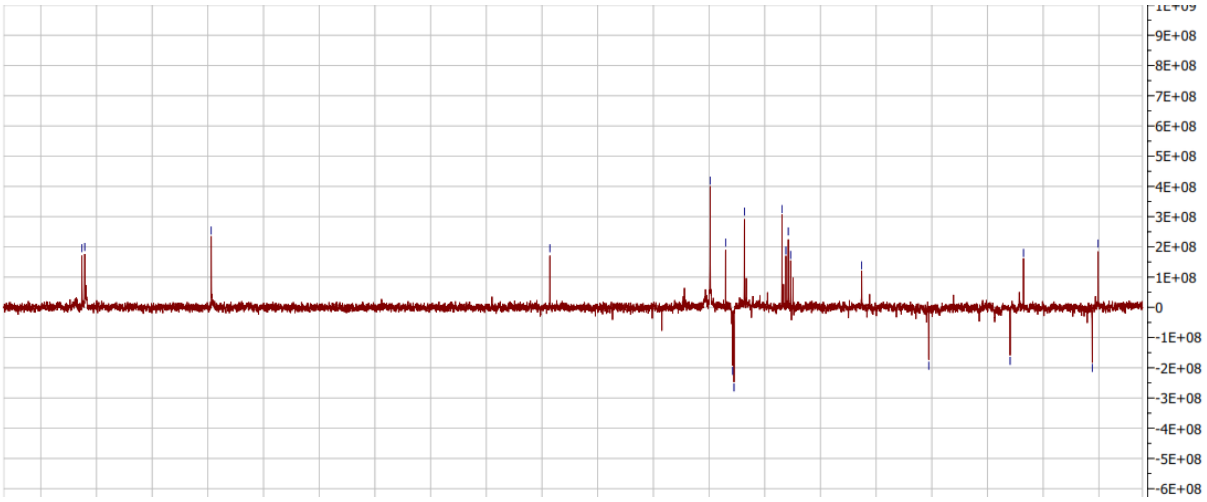

Figure S18: DEPT spectrum (125 MHz) of **4** in MeOD

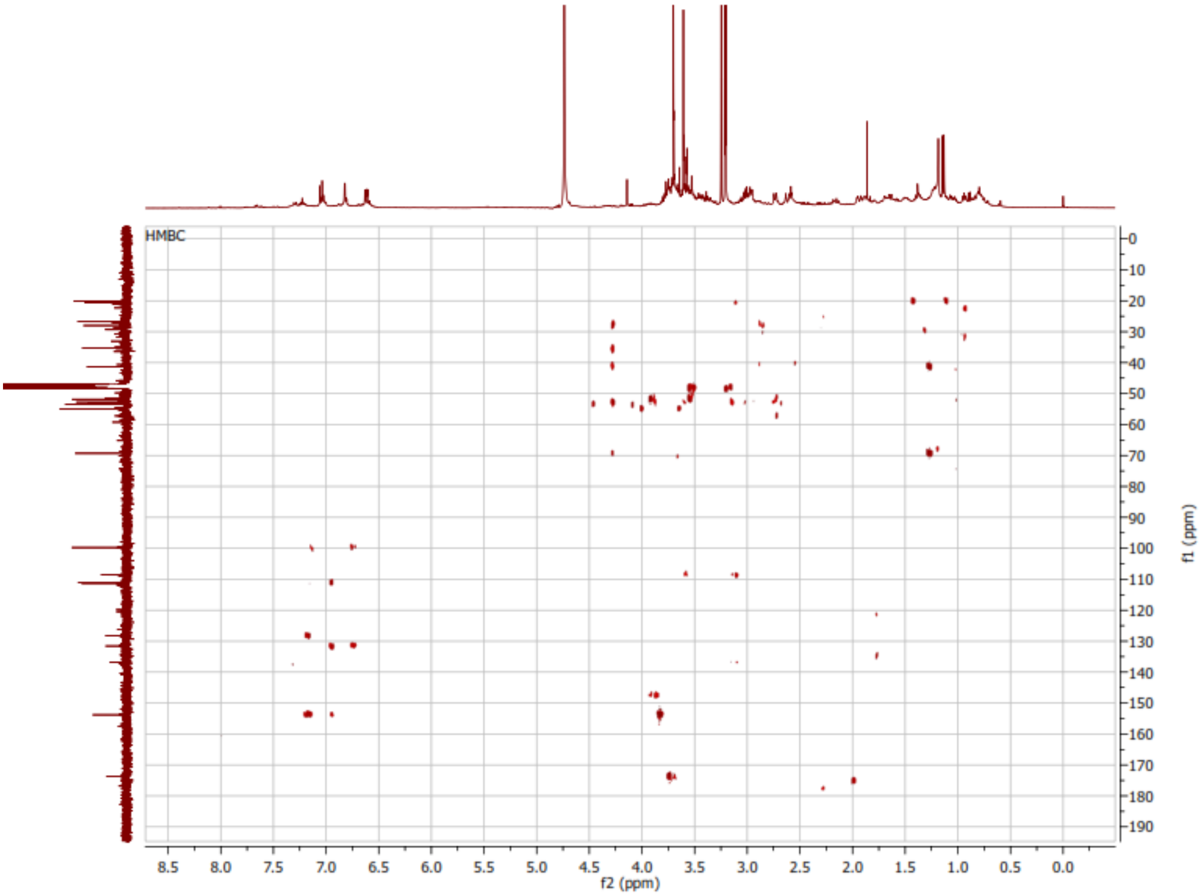

Figure S19: HMBC spectrum of **4** in MeOD

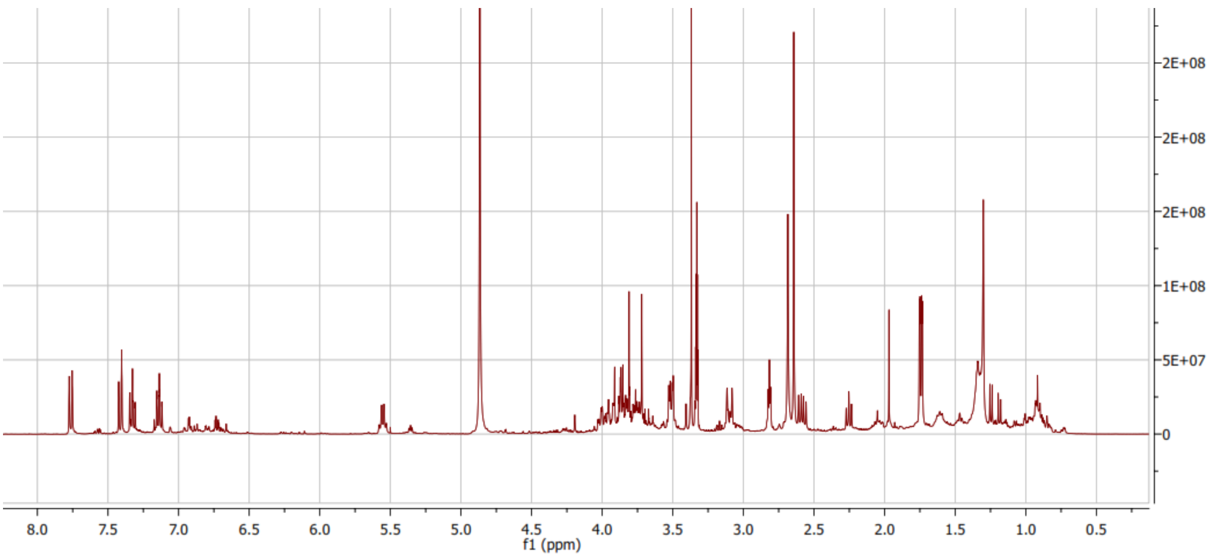

Figure S20: ^{1}H NMR spectrum (500 MHz) of **5** in MeOD

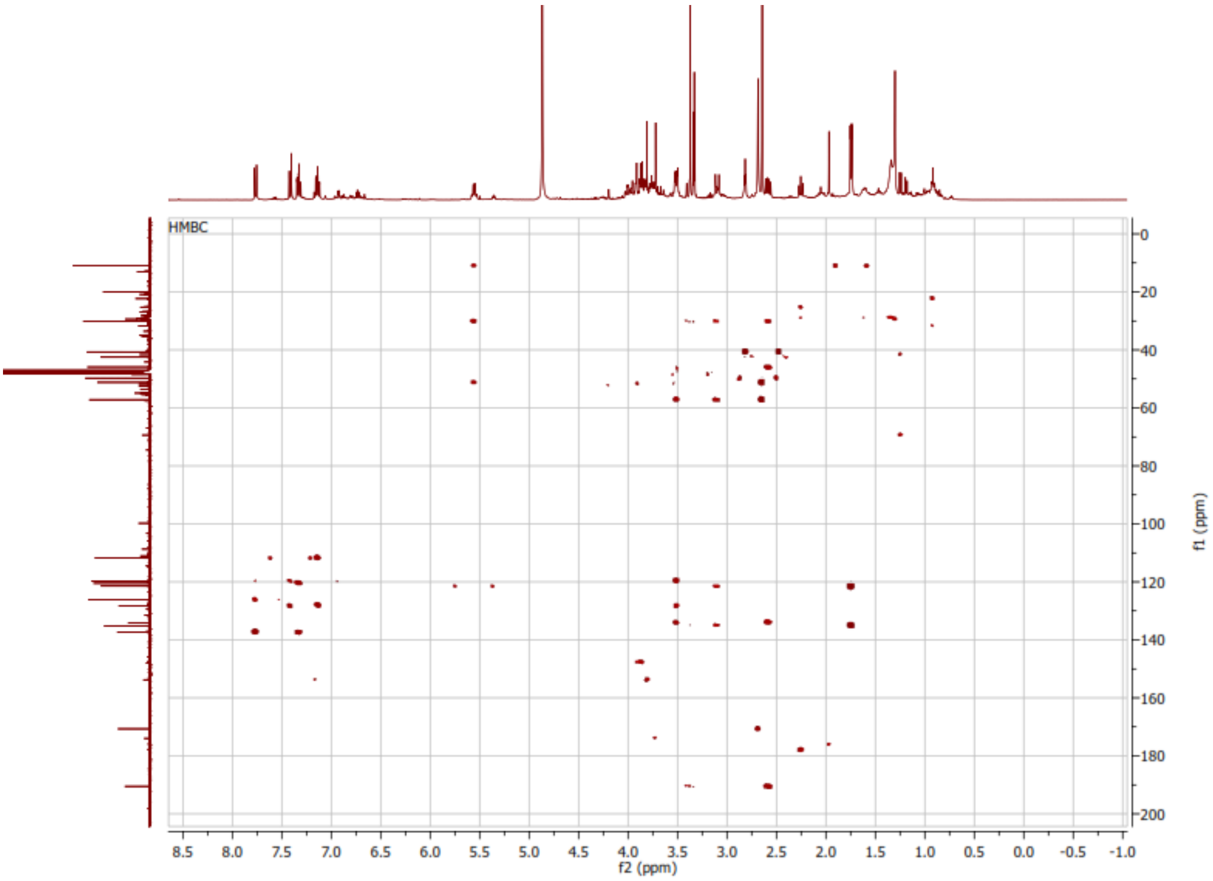

Figure S21: HMBC spectrum of **5** in MeOD

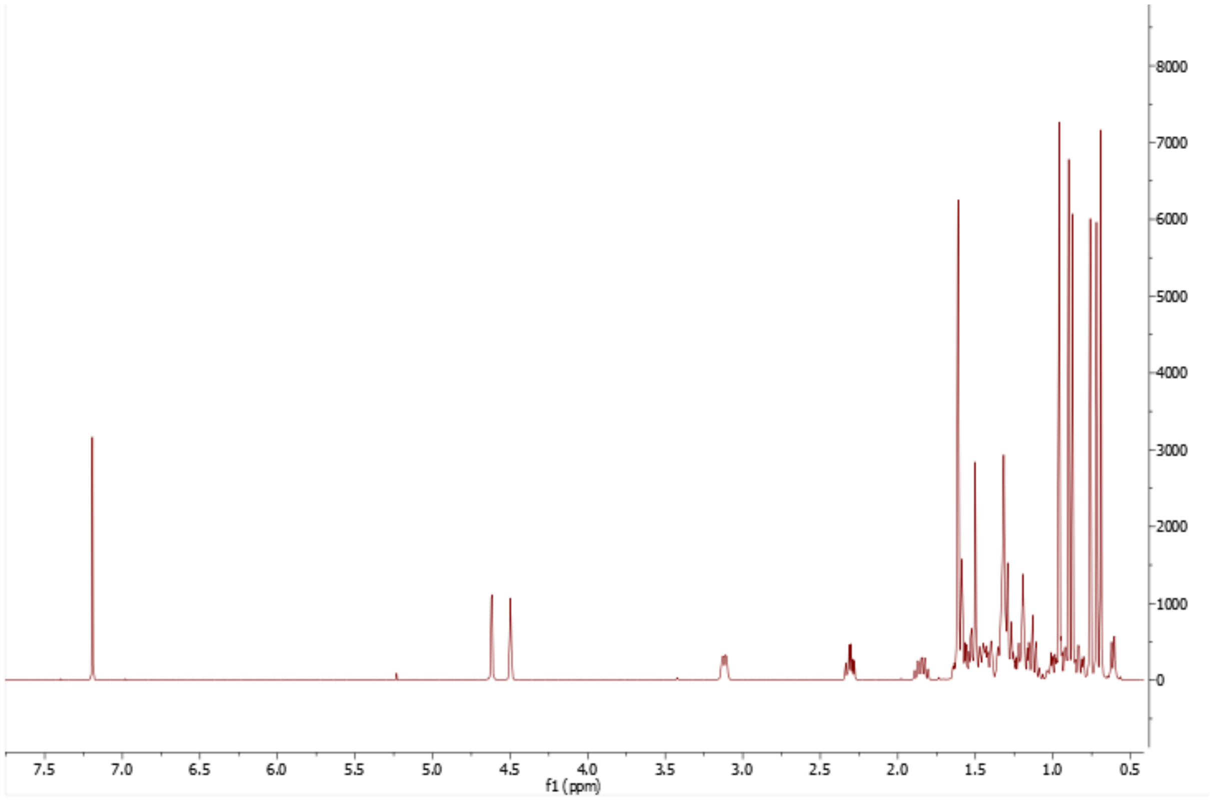

Figure S22: ^{1}H NMR spectrum (500 MHz) of **6** in $CDCl_3$

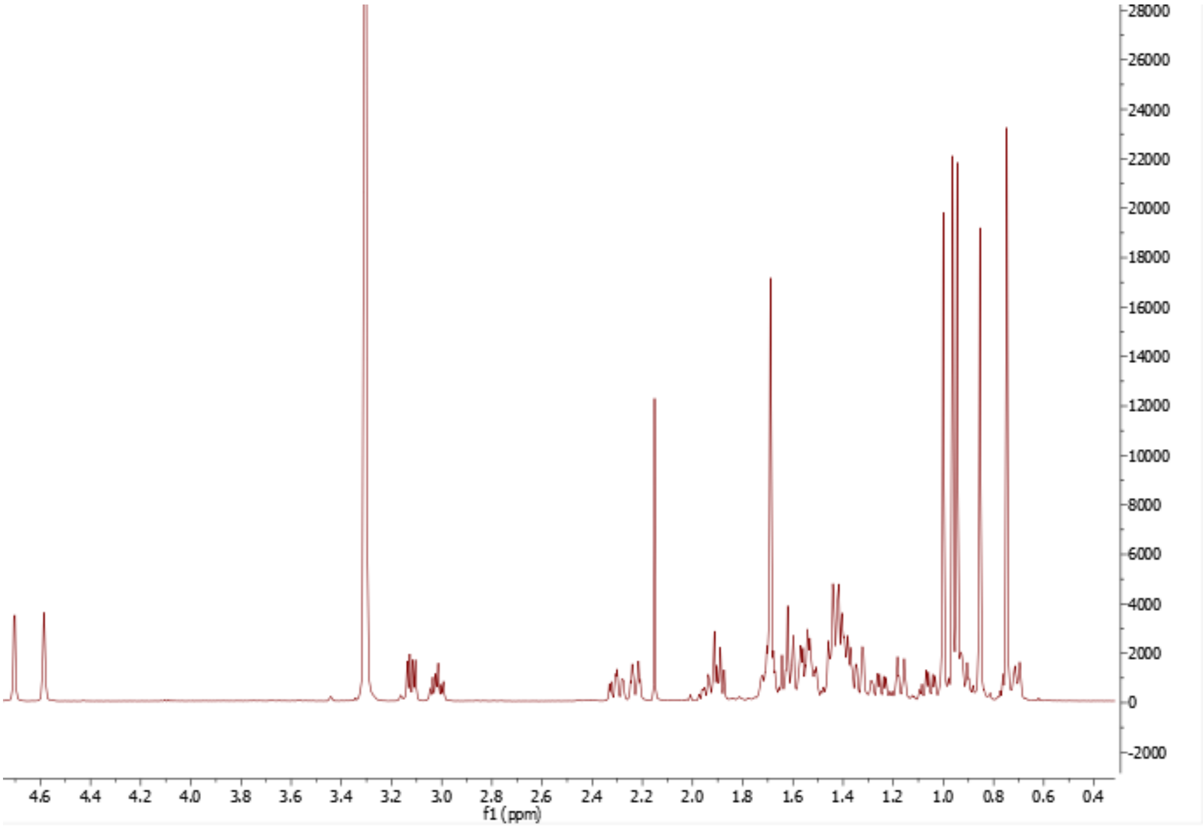

Figure S23: ^{1}H NMR spectrum (500 MHz) of **7** in MeOD

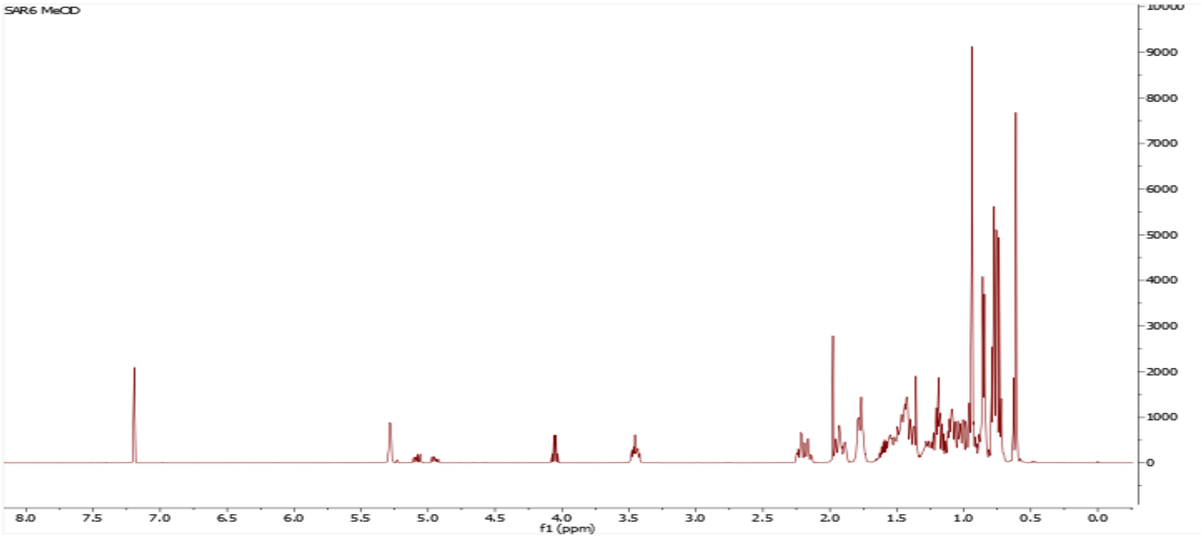

Figure S24: ^{1}H NMR spectrum (500 MHz) of **8** and **10** in $CDCl_3$

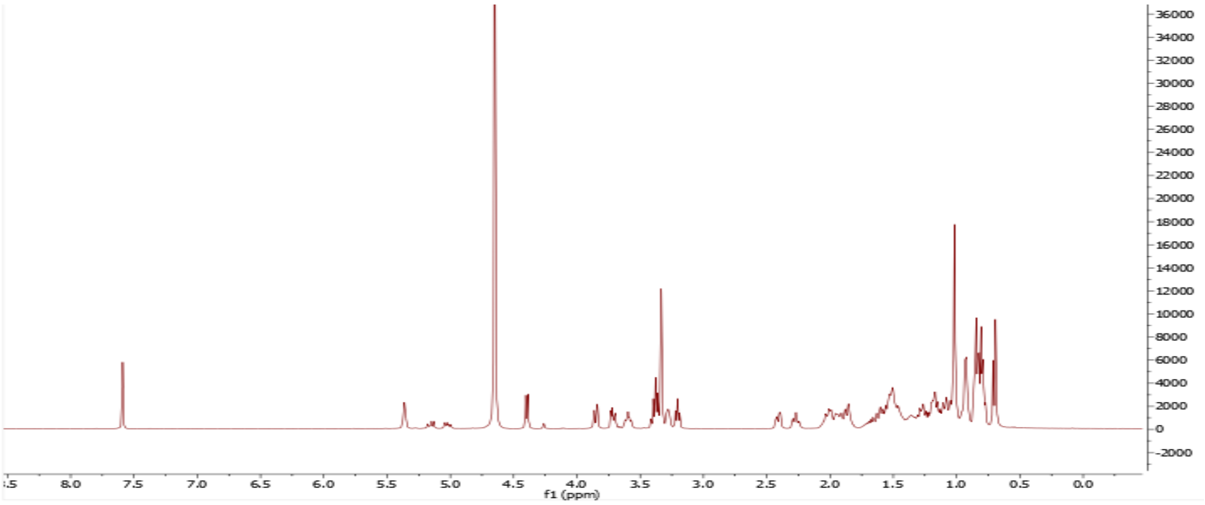

Figure S25: ^{1}H NMR spectrum (500 MHz) of **9** in $CDCl_3$/MeOD
